# The case against censoring of progression-free survival in cancer clinical trials – A pandemic shutdown as an illustration

**DOI:** 10.1186/s12874-022-01731-5

**Published:** 2022-10-05

**Authors:** Corinne Jamoul, Laurence Collette, Elisabeth Coart, Koenraad D’Hollander, Tomasz Burzykowski, Everardo D. Saad, Marc Buyse

**Affiliations:** International Drug Development Institute (IDDI), Av. Provinciale, 30 – 1340, Louvain-la-Neuve, Belgium

**Keywords:** Estimands, Censoring, Pandemic, Power, Bias, Progression-free survival

## Abstract

**Background:**

Missing data may lead to loss of statistical power and introduce bias in clinical trials. The Covid-19 pandemic has had a profound impact on patient health care and on the conduct of cancer clinical trials. Although several endpoints may be affected, progression-free survival (PFS) is of major concern, given its frequent use as primary endpoint in advanced cancer and the fact that missed radiographic assessments are to be expected. The recent introduction of the estimand framework creates an opportunity to define more precisely the target of estimation and ensure alignment between the scientific question and the statistical analysis.

**Methods:**

We used simulations to investigate the impact of two basic approaches for handling missing tumor scans due to the pandemic: a “treatment policy” strategy, which consisted in ascribing events to the time they are observed, and a “hypothetical” approach of censoring patients with events during the shutdown period at the last assessment prior to that period. We computed the power of the logrank test, estimated hazard ratios (HR) using Cox models, and estimated median PFS times without and with a hypothetical 6-month shutdown period with no patient enrollment or tumor scans being performed, varying the shutdown starting times.

**Results:**

Compared with the results in the absence of shutdown, the “treatment policy” strategy slightly overestimated median PFS proportionally to the timing of the shutdown period, but power was not affected. Except for one specific scenario, there was no impact on the estimated HR. In general, the pandemic had a greater impact on the analyses using the “hypothetical” strategy, which led to decreased power and overestimated median PFS times to a greater extent than the “treatment policy” strategy.

**Conclusion:**

As a rule, we suggest that the treatment policy approach, which conforms with the intent-to-treat principle, should be the primary analysis to avoid unnecessary loss of power and minimize bias in median PFS estimates.

## Introduction

Missing data can threaten the analysis and interpretation of clinical trials not only by decreasing statistical power, but also by introducing bias. In oncology, time-to-event endpoints play a key role in the assessment of treatment benefit in clinical trials. Although several endpoints may be affected in such trials, we wish to draw attention to progression-free survival (PFS), possibly the most frequently used primary endpoint in phase 3 trials in advanced cancer, notwithstanding critiques regarding its merits, for example, in comparison with overall survival [1]. Even though progression can be ascertained using clinical signs, symptoms, or levels of tumor markers, radiographic assessment remains the most used and preferred method on which PFS is based. Because radiographic progression can only be observed when imaging assessments occur, the actual time of progression is only known to have taken place after the former visit that showed no evidence of progression. Therefore, prolongation of the time between two assessments extends the apparent time to the event, thus making PFS particularly vulnerable to data missingness. For that reason, the FDA Guidance on Clinical Trial Endpoints for the Approval of Non-Small Cell Lung Cancer Drugs and Biologics [2] provides examples for analyses where events after two or more missed assessments are censored, arguing that a substantial number of missing tumor assessments can potentially over- or underestimate the treatment effect.

The concept of *estimands*, introduced recently in the International Conference on Harmonisation E9 (R1) guideline addendum, provides a common language to discuss intercurrent events in clinical trials [3]. Intercurrent events are events that have the potential to either preclude the observation of the variable of interest or affect its interpretation. The estimand framework creates an opportunity to define more precisely the target of estimation and ensures alignment between the scientific question and the statistical analysis. According to this framework, different estimands account for intercurrent events in a manner that addresses different questions. For example, in the context of PFS, possible intercurrent events include the typical situation of discontinuation from study treatment (e.g., due to toxicity) and/or initiation of another treatment prior to observing an event of progression or death [4]. At least three approaches can be considered in such cases [2]: (1) consider progression events when they occur, regardless of protocol treatment discontinuation and/or initiation of subsequent treatment; (2) consider such treatment change as an event; or (3) censor patients when they stop treatment and/or initiate subsequent treatment. In fact, these analyses address different questions, respectively: (1 – treatment policy) “Does the intent to use experimental treatment delay disease progression regardless of the treatment actually received?”; (2 – composite strategy) “Does the experimental treatment delay disease progression and/or initiation of subsequent treatment?”; and (3 – hypothetical strategy) “Does the experimental treatment delay disease progression in the absence of subsequent treatment?”.

The Covid-19 pandemic has greatly affected clinical trials for indications other than Covid-19. In oncology in particular, the pandemic has had a profound impact on health-care delivery and on the conduct of clinical trials [5–7]. Cancer patients are at higher risk of infection, complications, and death from Covid-19 than patients without cancer, and anticancer treatment may aggravate those risks [8–10]. Professional organizations have provided guidance for managing patients with cancer in the face of restrictions, inability and fear of access to treatment sites [11, 12], and regulatory agencies have issued guidelines for clinical trials during the pandemic [13, 14]. Various issues related to the pandemic can affect the conduct of clinical trials, and several aspects related to trial design, to patient enrollment and assessment, and to data collection have been addressed in the literature [5, 7, 15–21]. Such issues include staff limitations due to restrictions and infection; closure of sites, laboratories, and other providers; disrupted supply chain for medications; extended timelines for trial completion; and temporary or permanent interruption of a given trial. Attention has also been given to analytical issues, particularly with regards to missing data from missed visits, treatments and assessments, as well as to other types of protocol deviations [15, 17, 18, 22].

In the context of the Covid-19 pandemic, intercurrent events can be categorized as having a direct or an indirect impact on the trial [13]. Events that are caused primarily by Covid-19, such as those resulting in treatment discontinuation, use of additional medication, or death, have a direct impact and are directly covered by the estimand framework discussed above. Events that are due to overwhelmed health-care systems, regional lockdowns, or temporary follow-up interruptions due to logistic reasons, have an indirect impact through missing data. These latter events also need attention and can be addressed by the estimand framework.

In the context of PFS, one important indirect impact of the pandemic is delayed tumor assessment due to missed visits. In contrast with other endpoints that are measured at fixed time-points from randomization, assessing the impact of a calendar time event (such as a pandemic) on PFS probability curves is not straightforward. Each individual will be affected by the pandemic at a different time from their randomization, and the resulting impact on the study will depend on the PFS in each arm.

In this paper, we estimate the impact of a potential shutdown of health-care systems on the PFS analysis in a randomized trial, by considering two different strategies of handling a 6-month shutdown of all sites as an intercurrent event: a “treatment policy” strategy that ignores the event and a “hypothetical” strategy that censors patients with shutdown-related missed assessments followed by a PFS event (considering pandemic shutdown that prevents adequate tumor evaluation as an intercurrent event).

### Methods

#### Conceptual framework

We focused on the indirect impact of a potential shutdown of health-care systems that results in missed radiological assessments on the PFS analysis in a randomized trial. Using simulated data, we compared two approaches for handling the pandemic-related shutdown as an intercurrent event [3]: (1) the “treatment policy” approach, which consists in ascribing events—progression or death—to the time they are observed (acknowledging disease progression occurring during the shutdown period would be disclosed with delay); and (2) a “hypothetical” strategy, which consists in censoring patients whose events (progression or death) occur during the shutdown period at the last assessment before that period. The “treatment policy” strategy, ignoring occurrence of the intercurrent event, conforms with the intent-to-treat (ITT) principle, using all collected data and acknowledging the study was conducted in a non-perfect world, including pandemic-related logistic issues such as delayed assessments. On the other hand, censoring of time-to-event endpoints for an intercurrent event is widely used for targeting a hypothetical estimand. This “hypothetical” approach has been suggested as a way to account for the health-care-system disruptions related to the pandemic [23].

#### Simulations using fictitious trials

**Fig. 1:**
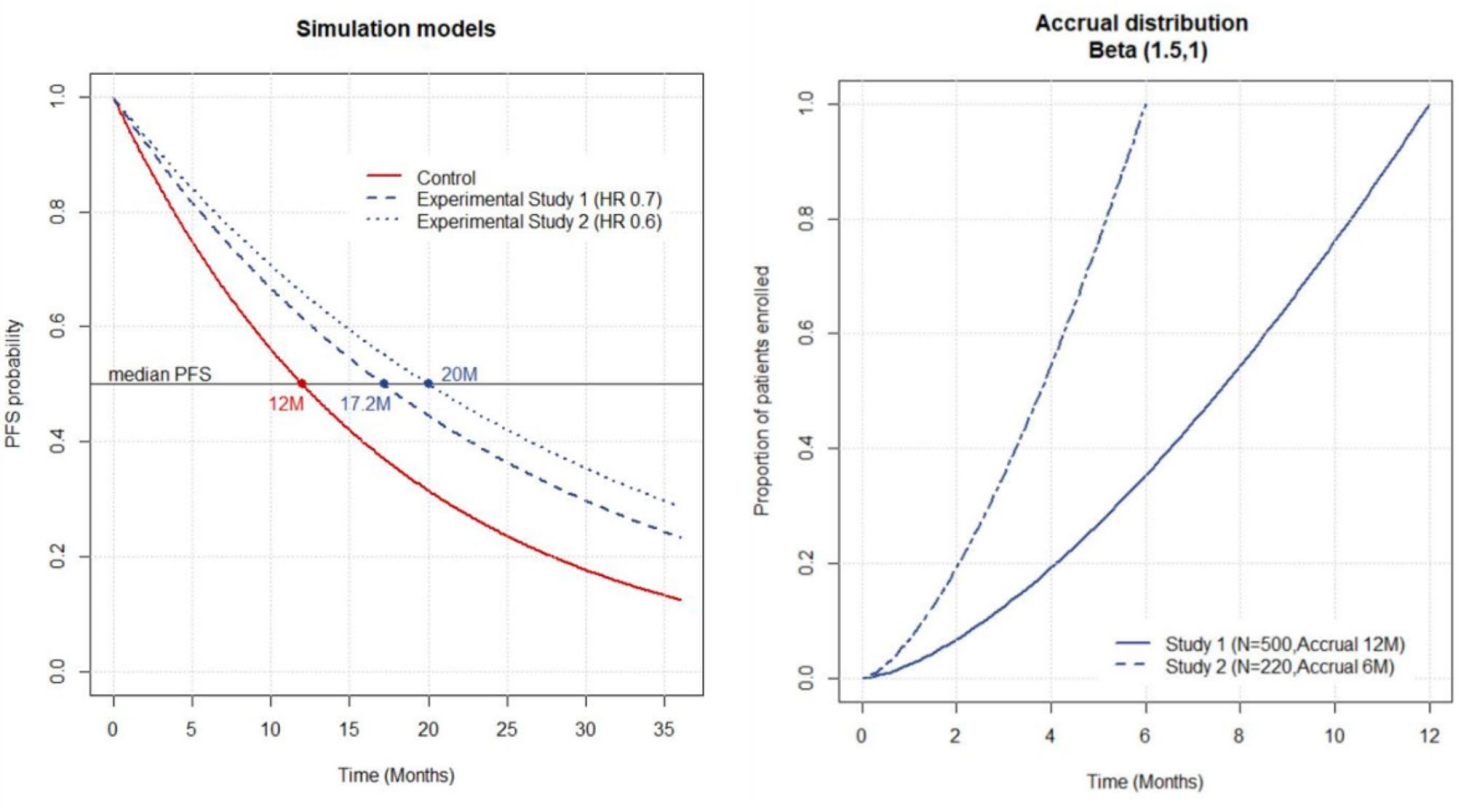
Progression-free survival and accrual distribution functions used in the simulations

We generated fictitious data for two scenarios. For Scenario 1, we simulated 10,000 clinical trials of 500 patients randomized in a 1:1 ratio to control or experimental treatment, assuming exponential distribution and proportional hazards (Fig. 1). A total of 331 events were required in order to have 90% power to detect a statistically significant difference in PFS between control and experimental arms with a 2-sided 5% type-I error, assuming a median PFS in the control arm of 12 months and a true hazard ratio (HR) of 0.70 (resulting in a median PFS of 17.2 months in the experimental arm). We simulated accrual of patients over a total period of 12 months using a beta distribution with parameters a = 1.5 and b = 1 to reflect a slower accrual at the beginning (Fig. 1). We assumed tumor scans were performed every 2 months, from randomization to documented progression or death (i.e., a PFS event).

In Scenario 2, we simulated 10,000 clinical trials of 220 patients randomized in a 1:1 ratio to control or experimental treatment, again assuming exponential distribution and proportional hazards. A total of 162 events were required to have 90% power to detect a statistically significant difference in PFS between arms with a 2-sided 5% type-I error, assuming a median PFS in the control arm of 12 months and a true HR of 0.60 (resulting in a median PFS of 20 months in the experimental arm). A 6-month accrual was assumed to follow the same beta distribution as in the previous scenario.

Based on simulated data, we computed the statistical power of the logrank test, estimated the HR using a Cox proportional hazards model, and estimated the median PFS in each arm using the Kaplan-Meier method. We then assumed the occurrence of a pandemic-related 6-month complete shutdown period during which patient enrollment stopped and no tumor scans could be performed in both scenarios. We considered several time intervals, with shutdown starting times ranging from 6 months to 24 months after beginning of accrual. At 6 months, scenario 2 implied completed accrual and scenario 1 implied that 35% of patients were recruited. For the latter scenario with shutdown during accrual, we assumed that enrollment of patients would be suspended during shutdown and delayed by 6 months. We assumed that all events occurring during shutdown were documented at the first tumor evaluation immediately after site reopening. For the sake of simplicity, we considered all events occurring during the shutdown period as progressions rather than progressions or deaths, acknowledging that in a real clinical trial, true death dates would be known and used as such with a “treatment policy” strategy. In all cases, we compared the indirect impact of the pandemic using the “treatment policy” and the “hypothetical” strategies. Analysis would be triggered once the required number of events required by design were documented. Two approaches were considered for the hypothetical estimand: (1) the total number of events accrued included all events, regardless of whether or not they would be censored at analysis level; (2) only uncensored events that would be used for the hypothetical estimand were taken into account in the monitoring of events, to ensure the analysis would be based on the required number of events. With the second approach, the power originally decreased by the censoring of events was tentatively restored at the price of a delayed time of analysis. Finally, we assessed results using an interval-censoring methodology.

We used the R-packages “survival” v3.2-10 for the logrank test, to fit the Cox model [24], and to estimate the median PFS; “interval” v1.1-0.7 for Turnbull Non-Parametric Maximum Likelihood Estimator of the survival function and weighted logrank test [25]; and “icenReg” v2.0.9 to fit the Cox model for interval-censored data without imputation of censored observations [26].

## Results

The results for the parameters of interest in the absence of the pandemic are shown in Table 1, confirming the design-related assumptions and reflecting the true parameters in the absence of the pandemic, which would be the target of a hypothetical estimand. The computed median PFS times with scans assumed to be performed every 2 months were slightly longer than the assumed true medians in the control and experimental arms, because of the well-known fact that progression dates in reality are left-censored, and are only a proxy for true progression dates when scans are performed at pre-specified intervals. The true median PFS times fell within the low and high values estimated by the interval-censoring method.

**Table 1:** Median values [95% range] in 10,000 simulated trials of median progression-free survival (PFS), hazard ratio, number of events and analysis time (in months), and power for the fictitious trials of Scenario 1 (N = 500 patients; target = 331 events) and Scenario 2 (N = 220 patients; target = 162 events) in the absence of the pandemic

Scenario	Parameter	Using exact time of events	Using tumor assessments every 2 months	Using tumor assessments every 2 months Interval-censoring method
1 (N = 500 patients; target = 331 events)	Median PFS, control	12.0 [10.0–14.4]	13.0 [11.0–16.0]	Low: 11.0 [8.0–14.0] High: 13.0 [11.0–16.0]
	Median PFS, experimental	17.2 [14.3–20.4]	18.0 [16.0–22.0]	Low: 16.0 [14.0–20.0] High: 18.0 [16.0–22.0]
	Hazard ratio	0.70 [0.56–0.87]	0.70 [0.56–0.87]	0.70 [0.56–0.87]
	Power	90.2%	90.0%	90.0%
	Number of events	331 [331–331]	331 [331–331]	331 [331–331]
	Analysis time	29.9 [27.4–32.6]	30.9 [28.4–33.6]	30.9 [28.4–33.6]
2 (N = 220 patients; target = 162 events)	Median PFS, control	12.0 [9.1–15.6]	13.0 [10.0–16.0]	Low: 11.0 [8.0–14.0] High: 13.0 [10.0–16.0]
	Median PFS, experimental	20.0 [15.1–25.9]	21.0 [16.0–27.0]	Low: 19.0 [14.0–25.0] High: 21.0 [16.0–27.0]
	Hazard ratio	0.60 [0.44–0.82]	0.60 [0.44–0.82]	0.6 [0.44–0.82]
	Power	89.8%	89.9%	89.8%
	Number of events	162 [162–162]	162 [162–162]	162 [162–162]
	Analysis time	33.6 [28.9–38.9]	34.6 [29.8–39.9]	34.6 [29.8–39.9]

**Fig. 2:**
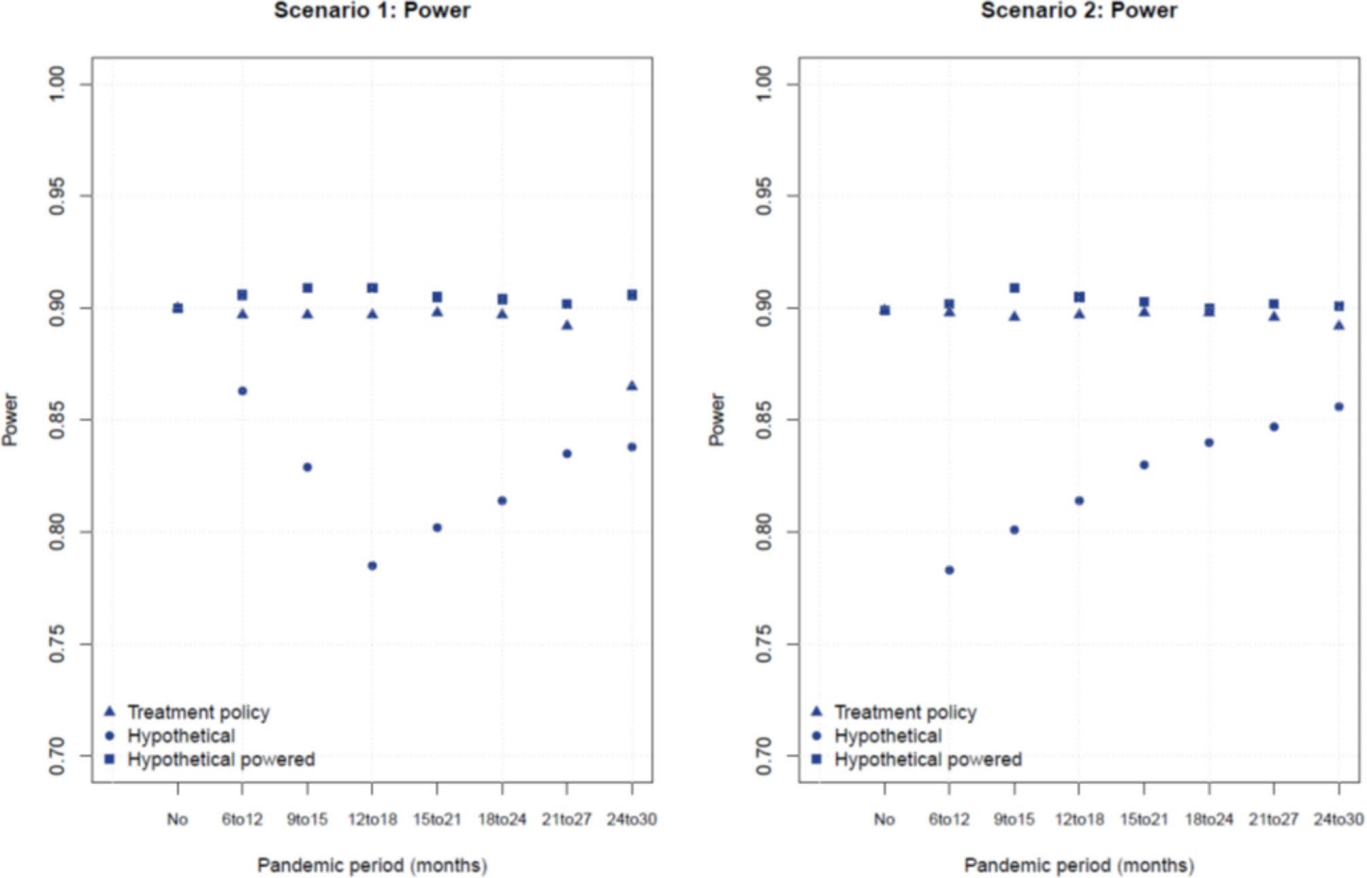
Power in each scenario as a function of pandemic period and strategy

Tables 2 and 3 display the parameters of interest in the presence of the pandemic, respectively for scenarios 1 and 2. Compared with the results in the absence of shutdown (Table 1), the “treatment policy” strategy slightly overestimated the median PFS in both arms. This is expected, given the assessments of disease progression is postponed to after the pandemic. The magnitude of this overestimation depended on the timing of the shutdown period. As an example, if the pandemic took place after sufficient follow-up was achieved and half of the patients had a documented progression in one arm, no impact on the median PFS was to be expected in that arm. With the “treatment policy” strategy, since events were still counted when detected upon site re-opening, the statistical power was not affected (Fig. 2). The power with compensation of events in the “hypothetical” strategy was slightly higher than the power with the “treatment policy” strategy, because with the former the additional events were not affected by the delay due to the pandemic. However, this increase in power came at the price of a delayed analysis, which should therefore be compared with a similarly delayed analysis using a “treatment policy” strategy.

**Table 2:** Median values [95% range] in 10,000 simulated trials of median progression-sree survival (PFS), hazard ratio, number of events and analysis time (in months), and power for the fictitious trial of Scenario 1 (N = 500 patients; target = 331 events), with 6-month shutdown periods occurring at various times

Shut-down period	Parameter	Treatment policy strategy	Hypothetical strategy	Hypothetical strategy, delayed analysis	Interval censoring
6 to 12 months	Median PFS, control	13.0 [11.8–16.0]	14.0 [12.0–18.0]	14.0 [12.0–18.0]	Low: 11.0 [8.0–14.0] High: 13.0 [11.8–16.0]
	Median PFS, experimental	18.0 [16.0–22.0]	20.0 [18.0–24.0]	20.0 [18.0–24.0]	Low: 16.0 [14.0–18.0] High: 18.0 [16.0–22.0]
	Hazard ratio	0.70 [0.56–0.87]	0.70 [0.55–0.88]	0.70 [0.56–0.88]	0.70 [0.56–0.87]
	Power	89.7%	86.3%	90.0%	89.0%
	Number of events	331 [331–331]	289 [277–301]	331 [331–331]	331 [331–331]
	Analysis time	35.3 [32.9–37.9]		41.3 [38.0–44.9]	35.3 [32.9–37.9]
12 to 18 months	Median PFS, control	14.0 [12.0–16.0]	18.0 [16.0–22.0]	18.0 [16.0–22.0]	Low: 10.0 [7.0–12.0] High: 14.0 [12.0–16.0]
	Median PFS, experimental	18.0 [16.0–22.0]	24.0 [20.0–28.0] 119 median estimates not reached	24.0 [20.0–28.0]	Low: 16.0 [12.0–20.0] High: 18.0 [16.0–22.0]
	Hazard ratio	0.70 [0.56–0.87]	0.69 [0.53–0.90]	0.69 [0.56–0.86]	0.70 [0.56–0.87]
	Power	89.7%	78.5%	90.9%	89.4%
	Number of events	331 [331–331]	225 [207–243]	331 [331–331]	331 [331–331]
	Analysis time	30.9 [28.4–33.6]		51.8 [45.7–60.8]	30.9 [28.4–33.6]
18 to 24 months	Median PFS, control	14.8 [13.1–16.9]	18.0 [14.0–20.0]	18.0 [14.0–20.0]	Low: 8.0 [8.0–11.0] High: 14.8 [ 13.1– 16.9]
	Median PFS, experimental	19.4 [16.7–22.0]	24.0 [20.0–28.0] 162 median estimates not reached	24.0 [20.0–28.0]	Low: 14.0 [11.0–18.0 ] High: 19.7 [16.8–22.0]
	Hazard ratio	0.70 [0.56–0.87]	0.70 [0.54–0.89]	0.70 [0.56–0.87]	0.70 [0.56–0.87]
	Power	89.7%	81.4%	90.4%	89.4%
	Number of events	331 [331–331]	253 [236–268]	331 [331–331]	331 [331–331]
	Analysis time	30.9 [28.4–33.6]		44.1 [39.7–49.5]	30.9 [28.4–33.6]
24 to 30 months	Median PFS, control	13.0 [11.0–16.0]	13.0 [11.0–16.0]	13.0 [11.0–16.0]	Low: 11.0 [9.0–12.0] High: 13.0 [11.0–16.0]
	Median PFS, experimental	19.0 [16.0–22.5]	20.0 [16.0–28.0] 238 median estimates not reached	22.0 [16.0–26.0]	Low: 12.0 [12.0–16.0] High: 20.0 [16.0- 23.7]
	Hazard ratio	0.71 [0.57–0.89]	0.70 [0.55–0.88]	0.70 [0.56–0.86]	0.69 [0.55–0.87]
	Power	86.5%	83.8%	90.6%	89.0%
	Number of events	331 [331–331]	274 [260–288]	331 [331–331]	331 [331–331]
	Analysis time	30.9 [30.0–33.6]		39.8 [36.2–44.1]	30.9 [28.4–33.6]

**Table 3:** Median values [95% range] in 10,000 simulated trials of median progression-free survival (PFS), hazard ratio, number of events and analysis time (in months), and power for the fictitious trial of Scenario 2 (N = 220 patients; target = 162 events), without pandemic (base case) and with 6-month shutdown periods occurring at various times

Shut-down period	Parameter	Treatment policy strategy	Hypothetical strategy	Hypothetical strategy, delayed analysis	Interval censoring
6 to 12 months	Median PFS, control	13.0 [10.5–16.0]	18.0 [16.0–22.0]	18.0 [16.0–22.0]	Low: 11.0 [4.0–14.0] High: 13.0 [10.5–16.0]
	Median PFS, experimental	21.0 [16.0–27.0]	26.0 [22.0–34.0] 67 median estimates not reached	26.0 [22.0–34.0]	Low: 19.0 [14.0–25.0] High: 21.0 [16.0–27.0]
	Hazard ratio	0.60 [0.44–0.82]	0.60 [0.41–0.86]	0.60 [0.43–0.81]	0.60 [0.44–0.82]
	Power	89.8%	78.3%	90.2%	89.2%
	Number of events	162 [162–162]	113 [101–124]	162 [162–162]	162 [162–162]
	Analysis time	34.6 [29.8–39.9]		80.8 [58.0–176.9]	34.6 [29.8–39.9]
12 to 18 months	Median PFS, control	14.9 [13.2–17.1]	18.0 [16.0–22.0]	18.0 [16.0–22.0]	Low: 8.0 [6.0–14.0] High: 14.9 [13.2–17.1]
	Median PFS, experimental	21.0 [16.5–27.0]	26.0 [22.0–34.0] 76 median estimates not reached	26.0 [22.0–34.0]	Low: 19.0 [10.0–25.0] High: 21.0 [16.5–27.0]
	Hazard ratio	0.60 [0.44–0.82]	0.60 [0.42–0.85]	0.60 [0.43–0.82]	0.60 [0.44–0.82]
	Power	89.7%	81.4%	90.5%	89.2%
	Number of events	162 [162–162]	125 [114–135]	162 [162–162]	162 [162–162]
	Analysis time	34.6 [29.8–39.9]		58.8 [47.1–79.1]	34.6 [29.8–39.9]
18 to 24 months	Median PFS, control	13.0 [10.0–19.6]	13.0 [10.0–22.0]	13.0 [10.0–22.0]	Low: 11.0 [8.0–12.0] High: 13.0 [10.0-19.6]
	Median PFS, experimental	22.0 [19.3–27.0]	26.0 [22.0–34.0] 68 median estimates not reached	26.0 [22.0–34.0]	Low: 15.0 [12.0–25.0] High: 22.0 [19.3–27.0]
	Hazard ratio	0.60 [0.44–0.82]	0.60 [0.42–0.84]	0.60 [0.42–0.84]	0.60 [0.44–0.82]
	Power	89.8%	84.0%	90.0%	89.2%
	Number of events	162 [162–162]	134 [124–143]	162 [162–162]	162 [162–162]
	Analysis time	34.6 [29.8–39.9]		50.0 [ 41.5–62.7]	34.6 [29.8–39.9]
24 to 30 months	Median PFS, control	13.0 [10.0–16.0]	13.0 [10.0–16.0]	13.0 [10.0–16.0]	Low: 11.0 [8.0–14.0] High: 13.0 [10.0–16.0]
	Median PFS, experimental	24.3 [16.0–28.0]	26.0 [16.0–32.0] 92 median estimates not reached	26.0 [16.0–34.0]	Low: 18.0 [14.0–21.0] High: 24.4 [16.0–28.0]
	Hazard ratio	0.60 [0.44–0.83]	0.60 [0.43–0.84]	0.60 [0.44–0.82]	0.60 [0.44–0.82]
	Power	89.2%	85.6%	90.1%	89.1%
	Number of events	162 [162–162]	141 [132–149]	162 [162–162]	162 [162–162]
	Analysis time	34.6 [30.0–39.9]		45.3 [37 − 9–54.8 ]	34.6 [29.8–39.9]

**Fig. 3:**
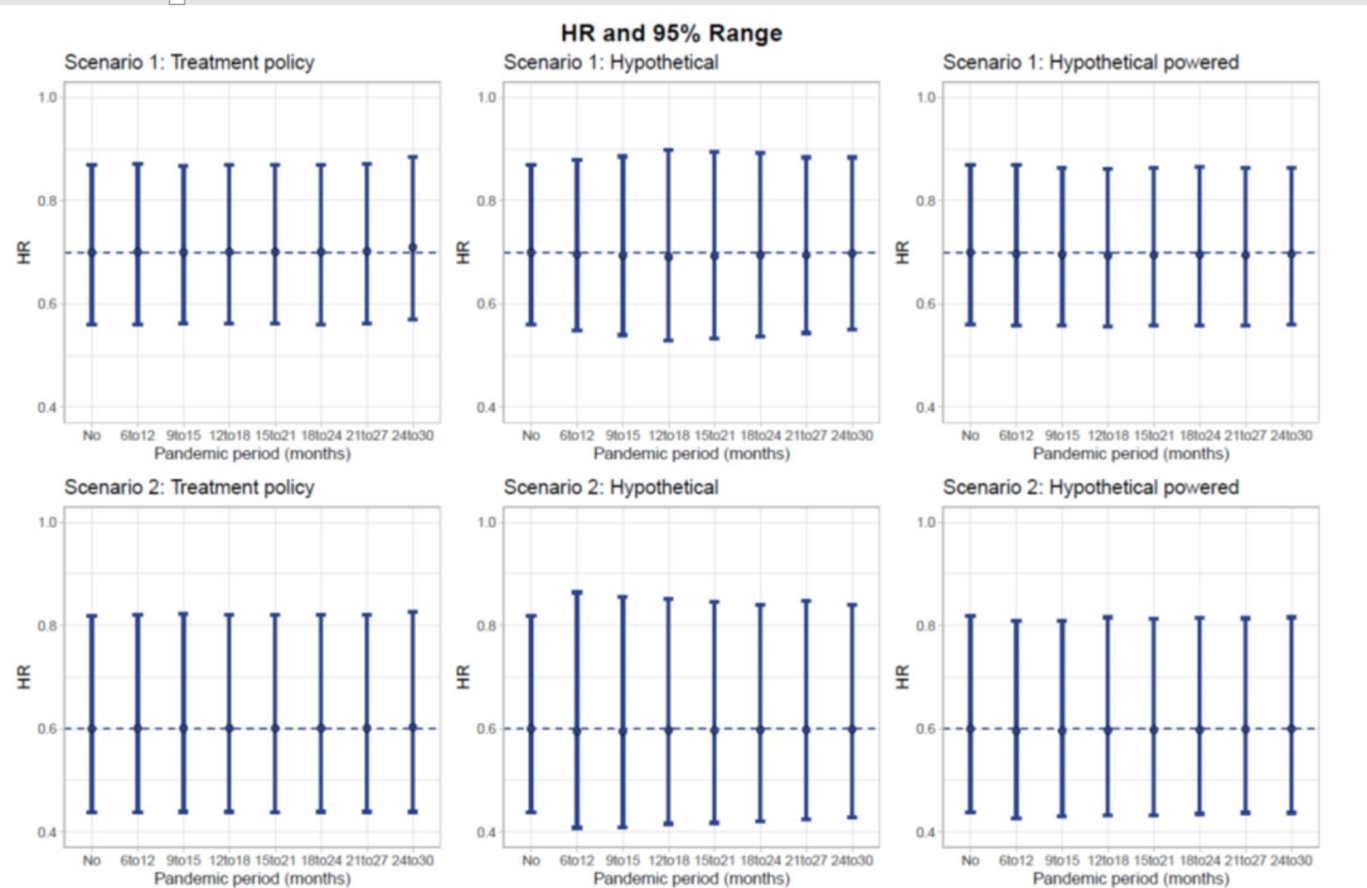
Hazard ratios and 95% range in each scenario as a function of pandemic period and strategy

**Fig. 4:**
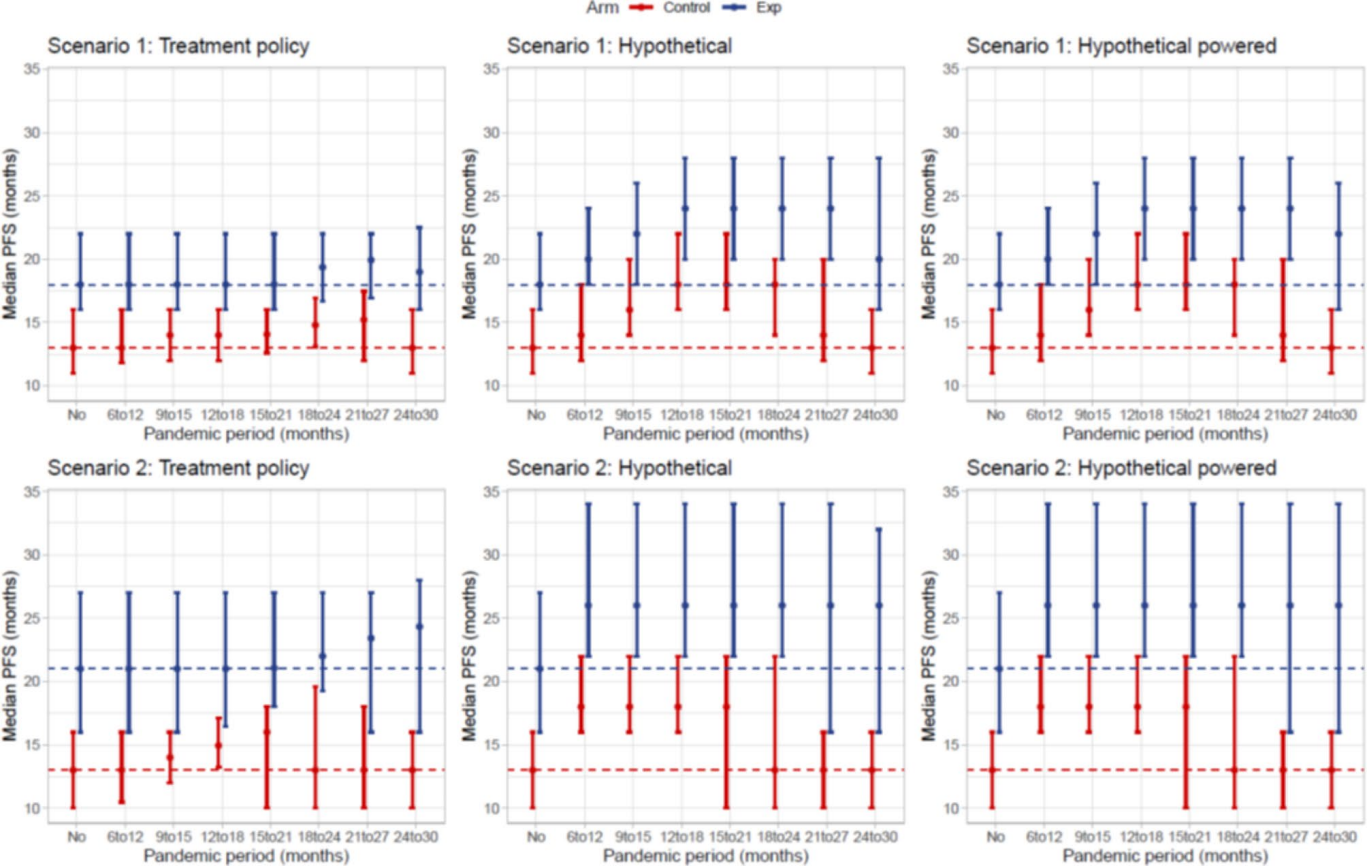
Median PFS in control (red) and experimental (blue) arms and 95% range in each scenario as a function of pandemic period and strategy

No appreciable impact on the estimated HR was observed, except for a slight dilution of the treatment effect with the “treatment policy” strategy in scenario 1 when the pandemic started 24 months after start of enrollment (HR = 0.72) (Fig. 3). In general, the pandemic had a greater impact on the analyses that use the “hypothetical” strategy. This method overestimated the median PFS times to a greater extent than the “treatment policy” strategy (Fig. 4). When the analysis was conducted at the same time as the “treatment policy” analysis, the median was not always reached as a result of the censoring (Tables 2 and 3). Moreover, the statistical power was decreased due to the smaller number of events (Fig. 2). Alternatively, the power for the hypothetical estimand could be maintained if more events were accrued to compensate for the censoring due to the pandemic. The study would then need to be prolonged by at least 6 months, and by up to 12 months (Tables 2 and 3). In scenario 2, when the pandemic affected a study that anticipated events in a large proportion of the patients (162/220), it might not be reasonably possible to catch-up on the number of events.

## Discussion

Censoring patients for intercurrent events has been a widely used strategy in the analysis of PFS. Before the introduction of the estimand framework, sensitivity analyses varied censoring rules, sometimes addressing different questions. Within the new framework these analyses represent different estimands rather than sensitivity analyses. Before the estimand framework, little attention was given to the fact that different censoring rules for intercurrent events (such as treatment discontinuation and initiation of subsequent treatment), performed to satisfy different stakeholders, actually addressed different clinical questions, or provided a biased estimate of the treatment effect of interest. As an example, censoring patients at the time of treatment discontinuation due to toxicity can bias the analysis in favor of the more toxic treatment in a randomized trial [4, 27].

For clinical trials designed before the Covid-19 pandemic and ongoing at the time it started, one of the main questions researchers have been asking is “Will the originally defined analysis (typically using a “treatment policy” strategy) correctly address the scientific question of interest”?

The oncology estimand working group, a cross-industry international collaboration, was established by the European Federation of Statisticians in the Pharmaceutical Industry as a European special-interest group for estimands in oncology, and was granted the official status of American Statistical Association scientific working group. In their publications, they have argued that the clinical trial objective should relate to a world without ongoing Covid-19 pandemic, including no major disruption of healthcare systems [23]. The censoring of time-to-event endpoints at the intercurrent event was suggested as a possible way to target a hypothetical estimand, even in the context of the Covid-19 pandemic-related healthcare system disruptions in PFS analysis [28].

Our simulated examples illustrate the indirect impact of a pandemic on results of ongoing trials when using a “hypothetical” strategy rather than the “treatment policy” strategy for handling a shutdown-related missing assessment situation. Our simulations show that the “hypothetical” strategy consisting of censoring those events documented immediately after the shutdown period has serious statistical implications. First, the loss of events due to censoring reduces the power of the statistical comparison unless the analysis time is delayed by several months. Second, this method results in a greater overestimation of the median PFS than the “treatment policy” strategy. Finally, as the maturity of the PFS curve and the median PFS are achieved at different times between treatment arms, the pandemic may affect different portions of the PFS curves in the two groups. For this reason, the imbalance in median overestimation can be more pronounced with the “hypothetical” strategy than with the “treatment policy” strategy. For all these reasons, the "treatment policy strategy" should remain the primary method of assessment. Interval-censoring methods, although not broadly used for primary analysis, would address the issue of unduly long intervals between scans. However, this more sophisticated method does not provide a point estimation of the median PFS.

Censoring a patient at the time of occurrence of an intercurrent event effectively estimates the PFS of patients in absence of that event as that of other patients that did not experience the intercurrent event at that time point and are in the same treatment arm. Therefore, the underlying assumption is that the arm A patient who experienced the intercurrent event at time *t* has the same PFS expectation from time *t* onward as the patients remaining on arm A at time *t* (‘non-informative censoring’). In many situations, e.g., censoring for switch to another treatment, the assumption does not hold. This may be a reasonable assumption, however, in the specific case of the shutdown of healthcare facilities during a pandemic. Nevertheless, as shown in our simulations, even when that assumption holds, censoring can result in a distortion of the treatment comparison when medians are used.

To evaluate whether the pandemic shutdown induced any bias in the evaluation of PFS, several sensitivity or supplemental analyses may be performed. As an example, an estimation of the times to the n^th^ visit (n = 1, 2, …, last) by treatment group might disclose an imbalance between treatment groups in the occurrence of delayed assessments induced by the pandemic.

It is important to note that each patient will be affected by the pandemic at a different time point in relation to their date of randomization. This difference between the time scale used for treatment comparison (time from randomization) and the calendar time stretches the portion of PFS curves affected by the pandemic shutdown to an extent that depends on the duration of accrual. As an example, if the 6-month pandemic shutdown starts 6 months after the end of a 12-month accrual period, we can only be reassured that the events observed within 6 months from randomization and after 24 months from randomization will not be affected by this shutdown period. As the true median PFS is assumed to be different under each treatment, the pandemic period, although not related to treatment, will affect each PFS curve in a different way, which may translate into an imbalanced overestimation of the median PFS between treatment groups. Indeed, when the shutdown starts before the median is reached in the experimental arm, but after sufficient follow-up is achieved and half of the patients have progressed in the control arm, only the median PFS in the experimental arm can be overestimated. This is illustrated, for example, by our findings related to Scenario 1 with the shutdown starting at 24 months and Scenario 2 with the shutdown starting at 21 and 24 months.

Under Scenario 1, results of the simulated study affected by a pandemic shutdown occurring 24 months from start of accrual illustrate that phenomenon, with a control median PFS estimate left unaffected but a treatment difference in median PFS of up to 10 months with the “hypothetical” approach (instead of the 5 months in the absence of a pandemic). Similarly, in Scenario 2, a treatment difference of 13 months (instead of the true difference of 8 months) was observed when the pandemic occurred 18 months from accrual start or later.

Our simulation studies were based on an exponential distribution assumption (in absence of the pandemic), which implies the assumption of proportional hazards. The hazard ratio, which is the appropriate measure of treatment effect under this assumption, is relatively unaffected by intercurrent events and censoring conventions. In situations commonly seen with immuno-oncology agents, such as delayed separation or crossing of the PFS curves (violating the proportional hazards assumption), and/or in settings for which a proportion of patients are expected to be cured, the impact of censoring for PFS because of the pandemic may affect the two arms in an even more unbalanced way than in our examples. These situations illustrate how censoring a time-to-event endpoint for intercurrent events that are completely independent from treatment might induce a bias in the treatment comparison. For this reason, in addition to the issues related to power loss due to massive censoring (which may not always be recovered), the practice of censoring for PFS, however well intended, is counterproductive. If interest truly focuses on a hypothetical estimand, methods based on causal inference can often be used instead, though in the presence of a pandemic affecting all patients, the opportunity for using such methods may be severely limited. Finally, the difference in median PFS is a statistically unstable and unreliable measure of treatment effect, and our results confirm that this statistic should generally not be used, regardless of the chosen estimand strategy [29, 30].

## Data Availability

The datasets used for the current study are available from the corresponding author on reasonable request. However, there are no raw data, as this work is based on simulations.
